# In vivo evaluation of decellularized skeletal muscle matrices for skeletal muscle repair: A systematic review

**DOI:** 10.1002/btm2.70009

**Published:** 2025-03-04

**Authors:** Ina Hennion, Charlot Philips, Chong Jiang, Nele Van De Winkel, Laurens J. Ceulemans, Lieven Thorrez

**Affiliations:** ^1^ Tissue Engineering Lab, Department of Development and Regeneration KU Leuven campus Kulak Kortrijk Belgium; ^2^ Department of Abdominal Surgery University Hospitals Leuven Leuven Belgium; ^3^ Department of Chronic Diseases and Metabolism, Lab of Respiratory Diseases and Thoracic Surgery (BREATHE) KU Leuven Leuven Belgium; ^4^ Department of Thoracic Surgery University Hospitals Leuven Leuven Belgium; ^5^ Leuven Intestinal Failure and Transplantation (LIFT) center University Hospitals Leuven Leuven Belgium

**Keywords:** acellular matrix, decellularization, preclinical models, recellularization, skeletal muscle regeneration, tissue engineering, volumetric muscle loss

## Abstract

Volumetric muscle loss is the significant loss of skeletal muscle volume beyond the innate regenerative capacity, resulting in functional impairment. The current standard of care combines muscle autografting with physical therapy but is often insufficient to reach full recovery. Decellularized skeletal muscle (DSM) provides an interesting alternative to repair volumetric muscle loss. The native structure and composition of the extracellular matrix in these acellular implants provide a blueprint for muscle regeneration. Moreover, DSM can be combined with cells to facilitate the regeneration of the skeletal muscle defect. This systematic review provides a complete and thorough overview of the state‐of‐the‐art applications and efficacy of DSM matrices in skeletal muscle repair in vivo, selected according to the Preferred Reporting Items for Systematic Reviews and Meta‐Analyses guidelines. Technical information on the different methods to create DSM implants and the implantation studies is provided. Moreover, details on the evaluation of the structural and functional regeneration of the muscle defect after implantation of the DSM are described. Results reveal a large heterogeneity in the analysis of regeneration upon DSM implantation. This heterogeneity makes it difficult to fully assess the efficiency of DSM to regenerate skeletal muscle, hampering further translation of this technique. Therefore, we suggest a multi‐level evaluation method to assess (i) muscle regeneration, (ii) vascularization, (iii) innervation of the regenerated muscle, and (iv) functional regeneration in a quantitative way.

**Translational Impact Statement**

Decellularized skeletal muscle (DSM) matrices hold great potential in restoring skeletal muscle loss. This addresses an unmet clinical need to regenerate functional skeletal muscle structure beyond the patient's native regeneration capacities. This systematic review provides a complete overview of the preclinical studies describing the application and engineering aspects of DSM tissue in skeletal muscle repair, showing the lack of a universal evaluation strategy. However, these insights are crucial in the further translation of DSM matrices to regenerate skeletal muscle defects in patients.

## 1 INTRODUCTION

Skeletal muscle tissue comprises about 40% of the total body mass and 50%–75% of all body proteins in humans, playing a crucial role in daily movement and in different metabolic processes.^1^ To execute this broad range of functions, skeletal muscle tissue is highly organized, containing aligned myofibers surrounded by different connective tissue layers. Moreover, skeletal muscle tissue is well vascularized and innervated.^1^, ^2^

Interestingly, skeletal muscle holds an inherent regenerative potential through a highly orchestrated process. Upon tissue damage and necrosis, debris will be removed during an initial inflammatory phase, in which neutrophils and pro‐inflammatory M1 macrophages are recruited.^3^, ^4^ Moreover, myofibroblasts deposit collagen as a scaffold for the regenerated myofibers later in the process.^5^ One to two days after injury, M2 macrophages become the predominant macrophage subtype, initiating the transition to the regenerative phase. In this phase, activated satellite cells (SCs) proliferate and differentiate to myoblasts, and fuse to form myofibers.^6^ During the remodeling phase, the deposited collagen is remodeled, and neovascularization occurs in the regenerated tissue. Finally, the regenerated skeletal muscle tissue acquires functionality and is innervated during the maturation phase.^3^, ^7^ This regenerative potential of skeletal muscle is of great importance to maintaining muscle homeostasis upon muscle injuries.

Volumetric muscle loss (VML) is the significant loss of skeletal muscle tissue that exceeds the endogenous regenerative capacity of skeletal muscle tissue. VML can lead to inflammation, tissue fibrosis and scar tissue formation, denervation, anatomical and biomechanical alterations, and ultimately to long‐term impairment and disability.^8^, ^9^, ^10^ Loss of skeletal muscle tissue can be caused by surgery, trauma, or disease.^9^ The incidence of VML in the general population is not systematically reported, and the large variety of VML causes and concomitant injuries makes it difficult to estimate the overall incidence. However, the available data indicate a substantial incidence of VML injuries. In a military setting, it is suggested that 77% of the patients with extremity trauma due to combat injuries experience VML.^11^ The projected lifetime disability costs for a soldier suffering VML are, on average 340,000–440,000 USD per individual, excluding medical costs.^11^ In the general population, VML is often caused by high‐energy trauma. Papakostidis et al. showed that 57% of the open tibialis shaft fractures in the general population were classified as type III open fractures with severe soft tissue damage.^12^

The current standard of care for VML consists of autologous tissue transfer, where a muscle flap is excised from an undamaged muscle and grafted into the injury site. Often, these grafting procedures are combined with rehabilitation therapy. Unfortunately, these treatments do not promote full functional recovery, and regeneration of the muscle is often not achieved.^9^ Moreover, the clinical applicability of autologous grafting is limited since morbidity can occur at the donor site, and the availability of donor tissue is limited.^13^ There is a clear need for alternative treatment options to regenerate skeletal muscle tissue after severe muscle loss in a clinical setting.

Decellularized skeletal muscle (DSM) matrices provide a promising graft to repair VML injuries. Decellularization aims to remove cellular material from donor tissue while minimally affecting the structure and composition of the extracellular matrix (ECM). The skeletal muscle ECM is a complex mesh‐network composed of collagen, glycoproteins, proteoglycans, and elastin and is an important regulator of cell fate and regeneration upon skeletal muscle tissue damage.^7^, ^14^, ^15^ Therefore, the preserved ECM structure and composition in tissue‐matched decellularized grafts support the remaining tissue and might promote cell migration and muscle regeneration after implantation.

Current research on DSM is primarily focused on identifying the optimal procedures to create DSM. The efficiency of the decellularization is evaluated through different in vitro analyses, assessing the removal of cellular material and the preservation of the ECM structure and composition.^14^ Moreover, the cytocompatibility of the scaffolds is often assessed in vitro. Initially, these in vitro analyses are of great importance to obtain a biocompatible acellular matrix with the intact ECM characteristics.^16^ However, skeletal muscle regeneration is a complex process that cannot be sufficiently assessed in vitro. The interaction of the regenerating skeletal muscle tissue with the immune system and vascular components is crucial for successful regeneration. Moreover, the mechanical and electrical stimulation in vivo might enhance myofiber alignment and maturation of the regenerated tissue.^10^ Since it is not possible to completely recapitulate these complex interactions in vitro, it is crucial to analyze the regenerative potential of DSM matrices in vivo. So far, no muscle‐derived acellular matrix has been implanted in human skeletal muscle defects. Thorough preclinical evaluations of DSM in animal models with skeletal muscle defects will be crucial to bridge this gap and bring DSM matrices to the clinical setting to help patients suffering from VML.

In a previous review, we highlighted the broad application potential of DSM for skeletal muscle tissue engineering. A few aspects of in vivo studies were described, and issues on the heterogeneity of these studies were raised.^7^ Building further on these observations, we now provide this state‐of‐the‐art systematic review on the efficacy of intact DSM matrices for skeletal muscle repair in preclinical models, with a specific focus on the employed methodology. First, technical aspects of the engineering process to generate DSM grafts are provided, including the decellularization and sterilization processes. Moreover, the possibilities of combining DSM matrices with cellular components are described. Next, the implantation procedures are discussed in depth, with information on the used animal models, transplantation type, defect size, defect site, and follow‐up time. Finally, and most importantly, this systematic review provides a critical overview of the different evaluation methods that are currently used to assess skeletal muscle regeneration upon DSM implantation. In this way, not only is the translational potential of DSM evaluated, but possible opportunities to accelerate research in the field are identified.

## 2 METHODS

### 2.1 Research objective

The aim of this systematic review is to provide an overview of all published research papers that evaluate the use of DSM to repair skeletal muscle defects in vivo. DSM was defined as a decellularized matrix that was created from skeletal muscle origin. Given the importance of ECM structure and organization in skeletal muscle regeneration, the systematic review focused on DSM of which the structure was kept intact upon implantation. DSM that were combined with a cellular component upon implantation was allowed.

### 2.2 Search strategy

To retrieve all eligible publications, a rigorous, systematic search was performed in both PubMed and Embase. The search terms included different synonyms regarding the inclusion criteria for (i) skeletal muscle, (ii) decellularized, and (iii) implantation. Moreover, some terms were excluded in the search query to limit the number of results that did not align with the scope of this review. Terms regarding cardiac muscle were excluded to omit research that is focused on cardiac muscle regeneration. “Dermal” and “nerve” were excluded to limit the number of papers that investigate a composite graft, including skeletal muscle with skin and/or nerve. Moreover, different bio‐fabrication terms were excluded to only retrieve papers in which the ECM structure was preserved upon implantation, aligning with the scope of this systematic review. Lastly, this systematic review focuses on original research papers, excluding reviews and systematic reviews.

PubMed was searched on January 19, 2024 with the following search term: *(“skeletal muscle”) AND (“decellularized” OR “decellularised” OR “acellular”) AND (“in vivo” OR “implantation” OR “transplantation” OR “repair” OR “regeneration”) NOT (“cardiac” OR “heart” OR “cardial” OR “dermal” OR “nerve”) NOT (“hydrogel” OR “bioinks” OR “bioink” OR “hydrogels” OR “electrospun” OR “electrospinning”) NOT (Review[Publication Type] OR Systematic Review[Publication Type])*.

Embase was searched on January 19, 2024 with the following search term: *(“skeletal muscle”/exp OR “skeletal muscle”) AND (“decellularized” OR “decellularised” OR “acellular”) AND (“in vivo”/exp OR “in vivo” OR “implantation”/exp OR “implantation” OR “transplantation”/exp OR “transplantation” OR “repair”/exp OR “repair” OR “regeneration”/exp OR “regeneration”) NOT (“cardiac”/exp OR “cardiac” OR “heart”/exp OR “heart” OR “cardial” OR “dermal” OR “nerve”/exp OR “nerve”) NOT (“hydrogel”/exp OR “hydrogel” OR “bioinks” OR “bioink”/exp OR “bioink” OR “hydrogels”/exp OR “hydrogels” OR “electrospun” OR “electrospinning”/exp OR “electrospinning”) NOT (review: it OR “systematic review”:it)*.

Search results were uploaded to Rayyan, an online tool that facilitates article selection. This allowed two researchers (IH, CJ) to perform an independent, blinded selection of the search results.^17^

### 2.3 Selection of relevant publications

**FIGURE 1:**
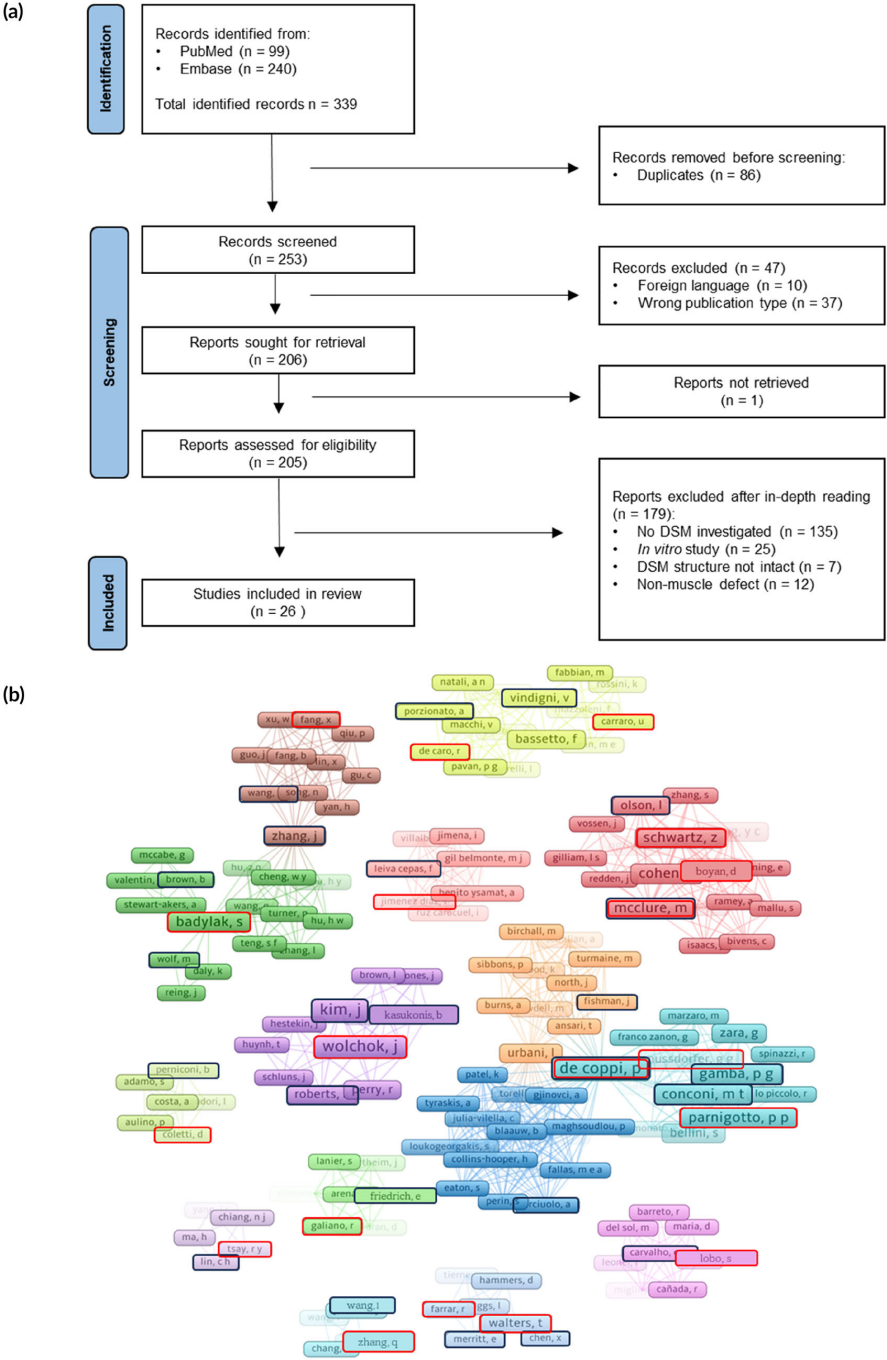
Inclusion of 26 research papers in the systematic review. (a) PRISMA flowchart of the selection process. (b) Clustering of all contributing authors of the 26 included publications using VOSviewer. Connecting lines based on co‐occurrence of the authors on the same included publication. Color based on cluster of authors, name size based on the number of occurrences of the author in the included publications. First authors of the included publications are indicated with a black box, last author of the included publications are indicated in a red box.

To provide a thorough and systematic search, the selection process was performed in accordance with the Preferred Reporting Items for Systematic Reviews and Meta‐Analyses (PRISMA) guidelines.^18^ Screening was performed by two researchers independently (IH and CJ) according to the following criteria. Inclusion criteria: all research papers investigating the implantation of intact DSM matrices in a skeletal muscle defect; recellularized DSMs were allowed. Exclusion criteria, in order of importance: publications in non‐English only; publication type other than research paper; no DSM matrix is investigated (e.g., non‐muscle ECM, synthetic material); in vitro study (without implantation of the DSM); structure of DSM is not intact (e.g., hydrogels, minced‐DSM, DSM‐paste); implantation site of the DSM is not within a skeletal muscle defect. For every retrieved publication that was excluded, one reason for exclusion was defined. After independent selection, the blind mode in Rayyan was switched off and the results were compared between the researchers. All conflicting selections were reviewed, and a final selection was determined after group discussion until consensus was achieved. The flowchart is graphically represented in Figure 1a.

### 2.4 Data extraction

Data extraction from the included papers was performed by one researcher and reviewed by a second researcher (IH and CP). Relevant data were extracted from all included studies, concerning:

- Decellularization and recellularization: decellularization method, decellularization agents, DSM size, DSM sterilization method, recellularization method, and recellularization cell type
- Implantation: donor animal, DSM source, DSM size, recipient animal, defect site, defect size, orientation of DSM, follow‐up time, use of immunosuppression, and adverse events
- Muscle regeneration: techniques used to assess muscle cell/fiber regeneration, immune cells and inflammation, ECM remodeling, and fibrous tissue formation
- Vascularization/innervation/functional regeneration: analysis methods

In case a certain element of the data extraction was not performed or was not mentioned in a specific publication, this was indicated as such in the table.

### 2.5 Likert scale

A 5‐point Likert scale was implemented based on the original evaluation of the authors in the reviewed paper regarding whether DSM matrices form a suitable repair option for skeletal muscle damage.

- Strongly agree (++): DSM forms a suitable repair option for skeletal muscle defects.
- Agree (+): DSM forms a suitable repair option for skeletal muscle defects; however, further research, improvement, or a better study design may improve the outcome.
- Neutral (0): uncertain/heterogenic results.
- Disagree (−): DSM does not form a suitable repair option for skeletal muscle defects; however, further research, improvement, or a better study design may improve the outcome.
- Strongly disagree (−−): DSM does not form a suitable repair option for skeletal muscle defects.

### 2.6 Data visualization

A network of the co‐authors of the included publications was generated using VOSviewer 1.6.20. All 159 co‐authors of the 26 included publications were included in the analysis. Data in multi‐layered pie charts was visualized using the Plotly package in Python 3.11.

## 3 RESULTS AND DISCUSSION

### 3.1 Inclusion of 26 studies in the systematic review

The initial search yielded a total of 339 publications, of which 240 were found through Embase and 99 through PubMed (Figure 1a). After the removal of 86 duplicates, the resulting 253 articles were screened for inclusion according to predefined criteria. An initial screening was based on publication type and language, resulting in 206 studies retained for full‐text screening, of which one article was inaccessible. After full‐text screening, 179 articles were excluded based on different exclusion criteria, as illustrated in the PRISMA flowchart. Ultimately, 26 articles were included in the systematic review and were subjected to in‐depth reading and data extraction.^19^, ^20^, ^21^, ^22^, ^23^, ^24^, ^25^, ^26^, ^27^, ^28^, ^29^, ^30^, ^31^, ^32^, ^33^, ^34^, ^35^, ^36^, ^37^, ^38^, ^39^, ^40^, ^41^, ^42^, ^43^, ^44^

The 26 included studies were published between 2002 and 2023 by different research groups. Clustering of the included publications based on author co‐occurrence using VOSviewer revealed 15 clusters of co‐authors of the included publications (Figure 1b). Some of the included publications are clustering closer together (e.g., Kim et al.,^29^ Kim et al.,^28^ Kasukonis et al.,^27^ and Roberts et al.^38^) with the same last author (Wolchok), while other included papers form isolated subclusters of authors.^28^

### 3.2 Heterogeneity in DSM matrices for implantation

In general, decellularization aims to remove all cellular material with minimal impact on the ECM to generate an acellular matrix with preservation of the structure and composition of the skeletal muscle ECM. This systematic review shows that there is a wide variety in the applied methods to achieve such a DSM matrix.

**FIGURE 2:**
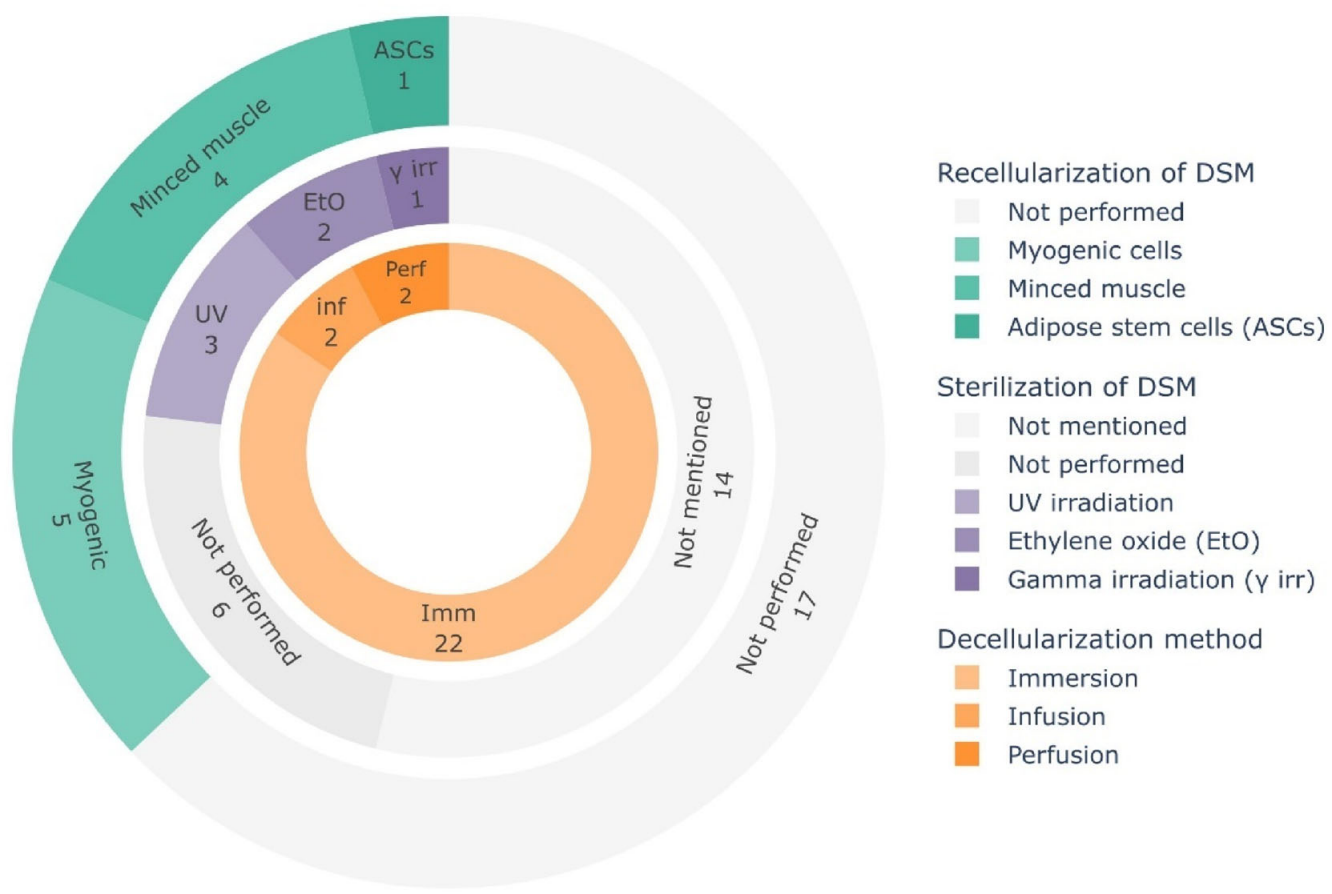
Overview of the decellularization, sterilization, and recellularization methods to create decellularized skeletal muscle matrices.

In general, three main decellularization techniques were applied to obtain DSM for implantation in skeletal muscle defects (Figure 2, Table 1). The majority of the studies (22 out of 26^19^, ^20^, ^21^, ^22^, ^23^, ^24^, ^25^, ^26^, ^29^, ^30^, ^31^, ^32^, ^33^, ^34^, ^35^, ^36^, ^37^, ^38^, ^40^, ^41^, ^42^, ^43^) used an immersion method, where tissue is submerged in alternating solutions. This method relies on the diffusion of the decellularizing agents into the tissue. Hence, immersion decellularization is intrinsically limited by the diffusion limit and could lead to unequal decellularization of the inner and outer parts of the tissue. To enhance the delivery of decellularizing agents into thicker tissue, the muscle can also be infused with decellularizing agents. Kasukonis et al.^27^ and Kim et al.^28^ decellularized the whole tibialis anterior muscle of rats by infusion (5 mL/hour) with a needle that was placed into the wide mid‐belly region of the muscle. Alternatively, as a third option, the native vasculature can be used to perfuse the muscle in a more homogeneous manner. Since the vasculature is designed to efficiently deliver oxygen and nutrients to the tissue, a perfusion decellularization method through the vasculature might be more efficient than an infusion method. Surprisingly, only two studies employed this technique to create DSM matrices for implantation experiments. Urciuolo et al.^39^ applied a perfusion method to decellularize the rat digitorum extensor longus, while Zhang et al.^44^ perfused part of the porcine abdominal muscles.

**TABLE 1:** Overview of the decellularization of skeletal muscle tissue to generate DSM matrices, and the terminal sterilization and recellularization of these matrices.

Decell method	Decellularizing agents	Terminal sterilization	Size DSM	Recellularization	Recellularization method	References
Immersion	Frozen; 0.02% trypsin/0.05% EDTA; 3% Triton X‐100; 4% deoxycholic acid; 0.1% peracetic acid/4% ethanol	Ethylene oxide gas	Not mentioned	Not performed	Not performed	Brown et al.^19^
Immersion	Frozen; 1% SDS; 5 mM EDTA/50 mM Tris +0.5% penicillin/streptomycin; 1% Triton X‐100 + 0.5% penicillin/streptomycin	Not mentioned	10 × 5 mm	Not performed	Not performed	Carvalho et al.^20^
Immersion	Frozen; 0.15% trypsin in DMEM; 10% fetal bovine serum (FBS) in DMEM; 0.3% Triton X‐100 + 2% ammonium	UV, overnight	Not mentioned: complete LD muscle?	Not performed	Not performed	Chen et al.^21^
Immersion	Twice: 4% SDC; 2000kU DNase I	Not mentioned	Not mentioned (15 × 15 mm for recellularization)	Autologous myoblasts; 80 × 10^6^cells/cm^2^; 24 h prior to implantation	Seeding on top	Conconi et al.^22^
Immersion	Twice: 4% SDC; 2000kU DNase I	Not mentioned	Not mentioned (30 × 30 mm for recellularization)	Myoblasts of male animals; 80 × 10^6^cells/cm^2^; 24 h prior to implantation	Seeding on top	De Coppi et al.^23^
Immersion	50 nM latrunculin B in DMEM; 0.6 M KCl; 1.0 M potassium iodide; 0.6 M KCl; 1.0 M potassium iodide; 1 KU/mL DNase I	UV, 30 min each side	Complete CAD muscle	EYFP labeled myoblasts; seeding 5 × 10^5^ cells per DSM; total 20 μL cell suspension injected in four places	Seeding on top + injection	Fishman et al.^24^
Immersion	0.25% SDS; 0.5 mM CaCl_2_ wash (only for SDSlow condition), 9 mg DNase I; PBS overnight (with negative pressure for SDSlow condition)	Not mentioned. All solutions used for the decell were sterile	Not mentioned	Not performed	Not performed	Friedrich et al.^25^
Immersion	Three times: 4% SDC; 2000 K units DNaseI	Not mentioned	Not mentioned: complete diaphragm?	Not performed	Not performed	Gamba et al.^26^
Infusion	1% SDS; incubation in 1 kU/mL; 1X penicillin/streptomycin	Not mentioned	Whole tibialis anterior muscle	Minced muscle autografts; 25% of created defect minced (±1 mm^3^); DSM rolled in minced muscle	Coating	Kasukonis et al.^27^
Infusion	1% SDS; incubation in 1 kU/mL; 1X penicillin/streptomycin	Not mentioned	Whole tibialis anterior muscle	Minced muscle autografts; 25% of created defect minced (±1 mm^3^); DSM rolled in minced muscle	Coating	Kim et al.^28^
Immersion	1% SDS; incubation in 1 kU/mL DNAse; 1X penicillin/streptomycin	Not mentioned	Not clearly mentioned: “smaller fragments”	Minced muscle autografts; 25% of created defect minced (±1 mm^3^); DSM rolled in minced muscle	Coating	Kim et al.^29^
Immersion	1% SDS; 0.5% Triton X‐100; DNase	Not mentioned	Not mentioned	Not performed	Not performed	Leiva‐cepas et al.^30^
Immersion	A. 0.1% trypsin/0.01% EDTA; PBS + sonication; 1% Triton X‐100; 0.1% aprotinin/PBS + sonication; DNase (50 U/mL)/RNAse (1 U/mL) in hypotonic buffer; PBS + sonication B. A. 0.1% trypsin/0.01% EDTA; PBS + sonication; 0.1 % SDS; 0.1% aprotinin/PBS with sonication; DNase (50 U/mL)/RNase (1 U/mL) in hypotonic; PBS + sonication C. 0.1% trypsin/0.01% EDTA; PBS + sonication; 0.1% SDS; 0.1% aprotinin/PBS + sonication; 1% Triton X‐100; 0.1% aprotinin/PBS 30 min + sonication; DNase (50 U/mL)/RNase (1 U/mL) in hypotonic buffer; PBS + sonication	Not mentioned	Not mentioned	Not performed	Not performed	Lin et al.^31^
Immersion	Frozen; multiple saline, detergent and disinfection soaks (proprietary method)	No sterilization: processed aseptically with disinfection soaks	Complete gastrocnemius muscle	Not performed	Not performed	McClure et al.^32^
Immersion	Frozen; multiple saline, detergent and disinfection soaks (proprietary method)	No sterilization: processed aseptically with disinfection soaks	Complete gastrocnemius muscle	Not performed	Not performed	McClure et al.^33^
Immersion	Chloroform; 2% SDS; 0.1 M Tris buffer solution of pH 9.0; PBS + 1% penicillin/streptomycin	UV, at least 12 h	Complete gastrocnemius muscle	Not performed	Not performed	Merrit et al.^34^
immersion	Frozen; multiple saline, detergent and disinfection soaks (proprietary method)	No sterilization: processed aseptically with disinfection soaks	Complete gastrocnemius muscle	Human myoblasts, human ASCs; 2 × 10^6^ cells/DSM scaffold; 24 h prior to implantation	Seeding on top	Olson et al.^35^
Immersion	1% SDS	No sterilization: decellularization solutions were sterile	Complete tibialis anterior muscle	Not performed	Not performed	Perniconi et al.^36^
Immersion	0.05% trypsin‐0.02% EDTA; 2% Triton X‐100‐0.8% NH_4_OH	No sterilization	15 × 20 mm	Not performed	Not performed	Porzionato et al.^37^
Immersion	1% SDS; 1kU/mL DNAse I and RNAse A; penicillin–streptomycin	Not mentioned	Not mentioned	Minced muscle autografts; 50% of the created defect minced and combined with DSM	Not specified	Roberts et al.^38^
Perfusion	A. 50 nM latrunculin B in DMEM; 0.6 M potassium chloride; 1 M potassium iodide for; 0.6 M potassium chloride; 1 M potassium iodide; perfusion DNase‐I in 1 M NaCl B. 4% SDC; 34 kU/ml DNase‐I in 1 M NaCl C. 0.25% SDS	Not mentioned	Intact lower limb	Not performed	Not performed	Urciuolo et al.^39^
Immersion	Three cycles of freezing (liquid nitrogen) and thawing (in a boiling water bath)	No sterilization	24 × 8 mm	Autologous satellite cells from; 10^6^ cells in 600 μL injected in the DSM	Injection	Vindigni et al.^40^
Immersion	Three times frozen (−80°C) and thawed (RT); 0.5 M NaCl; 1 M NaCl; 0.25% trypsin/EDTA; 1% Triton X‐100; DNase	Not mentioned	40 × 20 × 5 mm	Not performed	Not performed	Wang et al.^41^
Immersion	Twice (0.6 M KCl and 25 mM EDTA; 1 M KI); 1% Triton X‐100; 1% SDS; 70% ethanol; 25 mM Tris HCl	Gamma rays, 21 kGy, 24 h	50 × 50 × 5 mm	Not performed	Not performed	Wang et al.^42^
Immersion	Frozen (−80°C) and freeze‐dried; 2:1 solution of chloroform: methanol; 0.2% Trypsin/0.2% EDTA; 2% SDC; 1% Triton‐X 100; 0.1% peracetic acid/4% ethanol	Ethylene oxide, 16 h cycle at 50°C	2.25 mm thick sheets	Not performed	Not performed	Wolf et al.^43^
Perfusion	0.02% Trypsin/0.05% ethylene glycol tetraacetic acid via artery and vein; 0.1 % SDS via artery and vein; 1% Triton‐X 100 via artery and vein; 0.1% peracetic acid/4% ethanol via artery; DNase (40 U/mL)/α‐galactosidase (10 U/mL) via artery	Not mentioned	Complete distal half below umbilicus	Not performed	Not performed	Zhang et al.^44^

Besides the different decellularization techniques, a wide variety of decellularization protocols is applied to obtain DSM for implantation in skeletal muscle defects (Table 1). Commonly used decellularizing agents include chemical compounds such as peracetic acid, ethanol, ethylenediamine tetra‐acetic acid (EDTA), latrunculin B, and NaCl; detergents including Triton X‐100, sodium deoxycholate (SDC) and sodium dodecylsulfate (SDS) and enzymes such as DNase I and trypsin. A high variability is observed in the reviewed studies regarding the applied decellularizing agents, the concentrations, and treatment durations. Most commonly, there is a combination of a detergent (mainly SDC or SDS) with an enzymatic treatment (mainly DNase I or trypsin). Interestingly, McClure et al.^32^, ^33^ and Olson et al.^35^ collaborated with the Musculoskeletal Transplant Foundation to decellularize the skeletal muscle tissue by a proprietary protocol approved by the American Association of Tissue Banking and Food and Drug Administration. For future clinical translation, defined protocols approved by regulatory instances will be important to minimize variability in the decellularization process and allow further upscaling and automation.

The size of the DSM matrices is varying between different studies and—although this is a crucial element—is often not mentioned (Table 1). In general, the dimensions of the generated DSMs are still limited to a few millimeters, with a maximum of 50 × 50 × 5 mm by Wang et al.^41^ However, some publications describe the decellularization of the complete tibialis anterior muscle from mice^36^ and rats,^28^ the complete rabbit cricoarytenoideus dorsalis (CAD) muscle,^24^ the complete rat gastrocnemius muscle^32^, ^33^, ^35^ and a complete rat lower limb.^39^ Despite the larger sizes of these complete muscles, the dimensions of the DSMs are still below the dimensions that would be required for clinical translation. Hence, it is crucial to evaluate the regeneration upon implantation of large‐size DSM in large‐size defects in future studies.

A suitable DSM matrix should be successfully decellularized, generating a matrix without cellular components and preserved ECM. Moreover, the sterility of the final DSM matrix should be assured for the clinical applicability of the implant. Most studies either did not mention the use of any terminal sterilization steps (14 out of 26)^20^, ^22^, ^23^, ^25^, ^26^, ^27^, ^28^, ^29^, ^30^, ^31^, ^38^, ^39^, ^41^, ^44^ or did not perform any final sterilization (6 out of 26)^32^, ^33^, ^35^, ^36^, ^37^, ^40^ (Figure 2, Table 1). In some publications, it was explicitly mentioned that the applied decellularizing agents were sterile^36^ or that the tissue was handled in a sterile way throughout the procedure.^32^, ^33^, ^35^ Moreover, disinfection soaks in ethanol or antibiotics are included in some of the decellularization protocols to enhance the sterility of the DSM.^19^, ^20^, ^27^, ^28^, ^29^, ^32^, ^33^, ^34^, ^35^, ^38^, ^43^, ^44^ Terminal sterilization of the DSM was performed in 6 out of 26 studies, including the use of UV irradiation,^21^, ^24^, ^34^ ethylene oxide gas sterilization^19^, ^43^ and gamma irradiation^42^ (Figure 2, Table 1). It is clear that sterility is a key characteristic of the DSM from a translational point of view. However, different sterilization methods could also affect the integrity of the ECM proteins.^45^ Therefore, the influence of different sterilization methods on the structure and composition of the ECM in DSM matrices should be thoroughly assessed in vitro prior to implantation.

### 3.3 Recellularization of DSM to create biomimetic implants

The introduction of a cellular component in the DSM might enhance muscle regeneration upon implantation and might circumvent the difficulty of regenerating the core of large‐size defects. A recent systematic review and meta‐analysis of Greising et al.^46^ also suggested that an acellular biomaterial combined with stem and/or progenitor cells had the greatest treatment effectiveness in VML injuries.

In nine out of 26 studies, the DSM was combined with a cellular component prior to implantation^22^, ^23^, ^24^, ^27^, ^28^, ^29^, ^35^, ^38^, ^40^ (Figure 2, Table 1). In five studies, myogenic cells, including myoblasts and SCs, were seeded on top of the DSM.^22^, ^23^, ^24^, ^35^, ^40^ Based on the studies that were included in this systematic review, it remains unclear whether DSM combined with myoblasts or SCs results in superior muscle regeneration upon implantation compared to the DSM alone. Results of Conconi et al.^22^ and De Coppi et al.^23^ showed that muscle fibers were present in implanted DSM matrices that were seeded with myoblasts. However, Vindigni et al.^40^ showed that very few myoblasts and no muscle fibers were visible upon implantation of the recellularized DSM. From a developmental point of view, SCs and myoblasts are an evident choice for recellularization because of their importance in skeletal muscle regeneration. However, from a translational point of view, it might be more challenging to isolate sufficient numbers of SCs or myoblasts from the patient through a mildly invasive muscle biopsy.

Interestingly, Olson et al.^35^ recellularized DSM with a combination of human myoblasts and adipose‐derived stromal cells (ASCs). ASCs are an abundant source of multipotent mesenchymal stromal cells, which have been shown to support muscle regeneration.^47^ Previous in vitro studies also demonstrated the ability of ASCs to differentiate to myoblasts and fuse into myofibers.^48^, ^49^ Therefore, patient‐specific ASCs might be more convenient to use since they can be isolated from a less invasive liposuction. Olson et al.^35^ demonstrated that DSM alone, or DSM combined with either myoblasts or ASCs, are all capable of regenerating muscle fibers upon implantation. Moreover, ASCs improved levels of muscle regenerative markers compared to myoblasts.^35^

Surprisingly, none of the evaluated studies used induced pluripotent stem cells (iPSCs) for recellularization of DSM. This technology enables the generation of sufficient quantities of therapeutic patient‐derived cells. Advances in differentiating iPSCs toward muscle cells make them an attractive alternative for use in skeletal muscle regeneration.^50^ However, the lengthy and expensive manufacturing process to reprogram somatic cells, followed by the challenging differentiation into muscle cells, might hinder further clinical application.

The most commonly used technique to create these cellular DSM constructs is to seed the cells on top of the DSM scaffold 24 hours prior to implantation.^22^, ^23^, ^35^ To enhance the distribution of the cells into the DSM, Vindigni et al.^40^ injected SCs in the DSM scaffold (10^6^ cells in 600 μL). Alternatively, Fishman et al.^24^ generated a cellular construct by seeding myoblasts on top of the DSM scaffold and additionally injecting myoblasts in 3 places in the DSM. Next to the addition of myogenic cells or ASCs to create a cellular DSM construct for implantation, minced muscle autografts were added to the DSM in four studies.^27^, ^28^, ^29^, ^38^ Part of the skeletal muscle tissue that was excised to create the skeletal muscle defect (25% or 50%) was manually minced into small fragments of approximately 1 mm^2^. The DSM scaffold was rolled in this minced muscle containing a mixture of myogenic cells, supporting cells, and ECM components of the native skeletal muscle. The effectiveness of these combined grafts compared to empty DSM matrices remains unclear. Moreover, from a clinical perspective, the use of autologous minced muscle combined with DSM might not be feasible. The availability of donor tissue to generate the minced muscle is limited, and the procedure might induce donor site morbidity. Therefore, the use of minced muscle‐seeded DSM might not be able to overcome the major limitations of currently used autologous tissue transfer as a treatment for VML.

In most cases, autologous cells or autologous tissue were used to create the cellular component.^22^, ^27^, ^28^, ^29^, ^38^, ^40^ However, Olson et al.^35^ seeded the DSM with human myoblasts and human ASCs and implanted these cellular constructs in immunodeficient Foxn1^RNU^‐Nude (RNU) rats. No rejection of the implanted cellular xenografts was described. Moreover, De Coppi et al.^23^ seeded DSM with myoblasts derived from male rats and implanted the constructs in female rats in the absence of immunosuppressive therapy. No inflammation or disintegration of the implanted cellular DSM was reported. Interestingly, this approach allows tracking the migration of cells derived from the cellular DSM scaffold in vivo by detecting the Y chromosome with fluorescent in situ hybridization and qPCR analysis (sex‐determining region Y gene). As an alternative to track cellular migration, Fishman et al.^24^ combined the DSM with labeled myoblasts obtained from mice ubiquitously expressing the enhanced yellow fluorescent protein under the control of a β‐actin promoter.

### 3.4 Implantation of DSM in skeletal muscle defects

#### 3.4.1 Small animal models to implant DSM matrices without immunosuppression

**FIGURE 3:**
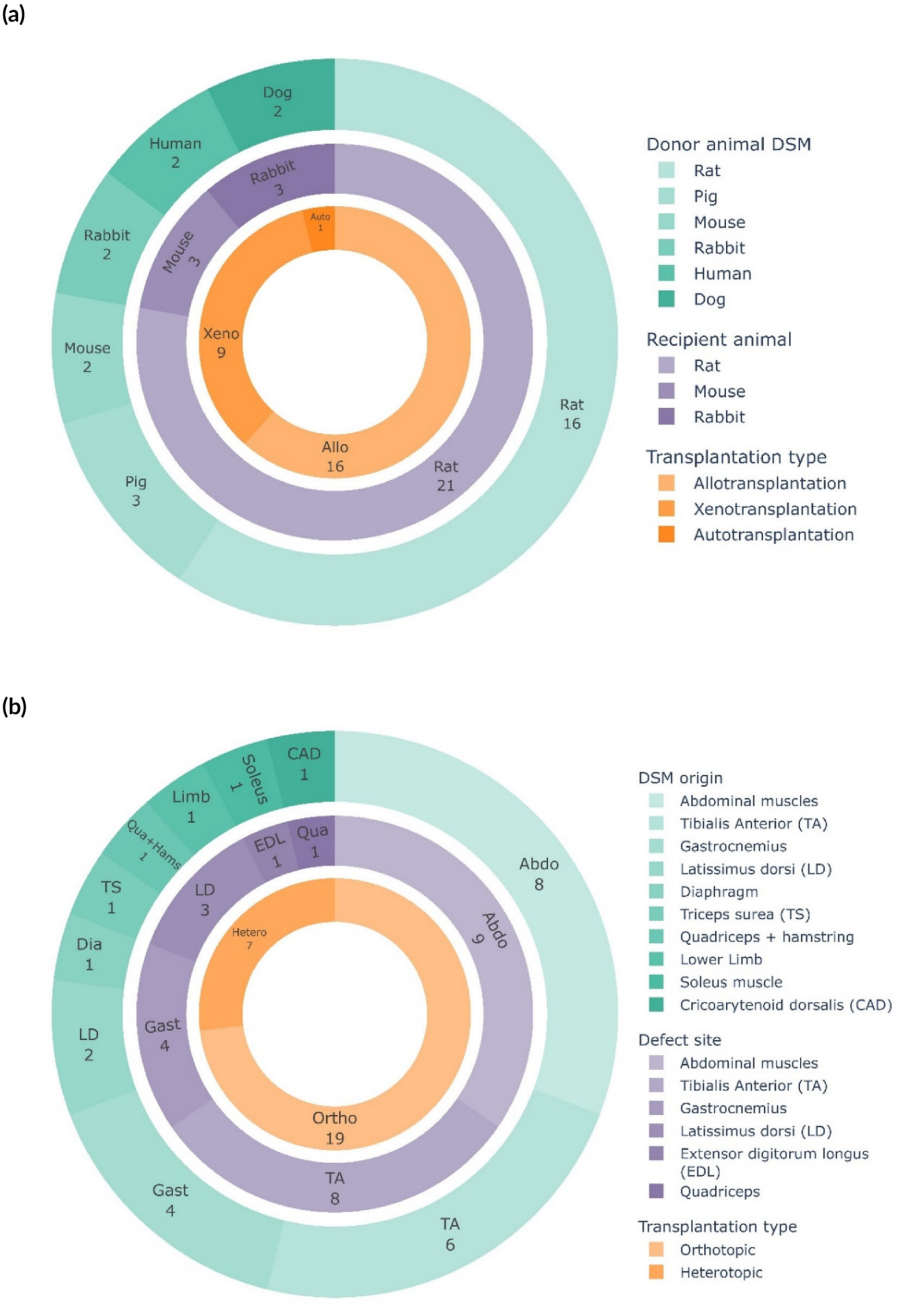
Implantation of decellularized skeletal muscle matrices in skeletal muscle defects. (a) Used donor and recipient animals. (b) DSM origin and skeletal muscle defect site.

To accurately assess the capacity of DSM grafts to regenerate skeletal muscle defects, the DSM grafts should be implanted in a skeletal muscle defect. Currently, DSM matrices have only been implanted in smaller animal models (Figure 3a, Table 2). Rodents are the most frequently used recipient animals to evaluate skeletal muscle repair. Mostly (21 out of 26 studies), rats have been used, including Sprague–Dawley rats,^19^, ^22^, ^24^, ^25^, ^32^, ^33^, ^38^, ^42^, ^43^, ^44^ Wistar rats,^20^, ^30^, ^40^ Lewis rats,^21^, ^23^, ^34^ Fischer 344 rats,^27^, ^28^, ^29^, ^41^ and RNU rats.^33^, ^35^ In addition, mice were used in three studies as recipient animals.^31^, ^36^, ^39^ These small, easy‐to‐handle, and lower‐cost animal models are well suited for initial in vivo studies, where different groups with a larger sample size are compared to further optimize the creation of DSM matrices for implantation. However, caution should be taken when translating findings regarding muscle regeneration in these small rodents to the clinical setting in humans, since it has been suggested that not all mechanisms regulating mouse SC activation are conserved in human SCs.^51^ The largest recipient animals thus far used are New Zealand White rabbits.^26^, ^37^, ^42^

**TABLE 2:** Implantation of decellularized skeletal muscle matrix in skeletal muscle defects.

Donor	Muscle origin	DSM size	Recipient	Defect site	Defect size	Follow‐up	Immuno‐supression	Tx type	Tx type	Reference
Rat: Sprague–Dawley	Ventral abdominal wall	Not mentioned	Rat: Sprague–Dawley	Ventrolateral abdominal muscles (partial thickness)	10 × 10 mm	3, 7, 14, and 28 days	Not mentioned	Allogenic	Orthotopic	Brown et al.^19^
Rat: Wistar	Tibialis anterior	10 × 5 mm	Rat: Wistar	Tibialis anterior	Not applicable (skeletal muscle laceration)	3, 15, and 45 days	Not mentioned	Allogenic	Orthotopic	Carvalho et al.^20^
Rat: Lewis	Latissimus dorsi muscle	Not mentioned: complete LD muscle?	Rat: Lewis	Latissimus dorsi	8 × 12 mm	8 weeks	Not mentioned	Allogenic	Orthotopic	Chen et al.^21^
Rat: Sprague–Dawley	Abdominal muscles	Not mentioned (15 × 15 mm for recellularisation)	Rat: Sprague–Dawley	Abdominal muscles: between interal and external oblique muscles	No defect created	12, 30, and 60 days	Not used	Allogenic	Orthotopic	Conconi et al.^22^
Rat: Lewis	Abdominal muscles	Not mentioned (30 × 30 mm for recellularisation)	Rat: Lewis	Abdominal wall: full‐thickness	2 mm^2^	1, 3, and 9 months	Not used	Allogenic	Orthotopic	De Coppi et al.^23^
Rabbit: New Zealand white	Cricoarytenoid dorsalis (CAD) muscle	Not mentioned (6 mm biopsy punch for recellularisation)	Rat: Sprague–Dawley	Tibialis anterior	4 mm diameter biopsy punch	2 and 4 weeks	Not used	Xenogenic	Orthotopic	Fishman et al.^24^
Rat: Sprague–Dawley	Abdominal muscles	Not mentioned	Rat: Sprague–Dawley	Latissimus dorsi	10 mm biopsy punch	30 days	Not mentioned	Allogenic	Heterotopic	Friedrich et al.^25^
Rabbit: New Zealand white	Diaphragm	Not mentioned: complete diaphragm?	Rabbit: New Zealand white	Abdominal wall: external oblique muscle	30 × 30 mm	6, 40, and 90 days	Not used	Autogenic	Heterotopic	Gamba et al.^26^
Rat	Tibialis anterior	Whole tibialis anterior muscle	Rat: Fischer 344 (3 months old and 18 months old)	Tibialis anterior	8 mm diameter biopsy punch, 3 mm deep	12 weeks	Not mentioned	Allogenic	Orthotopic	Kasukonis et al.^27^
Rat: Sprague–Dawley	Tibialis anterior	Whole tibialis anterior muscle	Rat: Fischer 344	Tibialis anterior	8 mm diameter biopsy punch, 3 mm deep	12 weeks	Not mentioned	Allogenic	Orthotopic	Kim et al.^28^
Human	Tibialis anterior	Not clearly mentioned: “smaller fragments”	Rat: Fischer 344	Tibialis anterior	8 mm diameter biopsy punch, 3 mm deep	8 weeks	Not mentioned	Xenogenic	Orthotopic	Kim et al.^29^
Rat	Soleus muscle	Not mentioned	Rat: Wistar	Tibialis anterior	6 mm × 5 mm cylindrical fragment	60 days	Not mentioned	Allogenic	Heterotopic	Leiva‐cepas et al.^30^
Mouse: ICR	Latissimus dorsi muscle	Not mentioned	Mouse: ICR?	Latissimus dorsi	10 × 20 mm	12, 20, and 30 days	Not mentioned	Xenogenic	Orthotopic	Lin et al.^31^
Rat: Sprague–Dawley	Gastrocnemius	Complete gastrocnemius muscle	Rat: Sprague–Dawley	Gastrocnemius	10 × 10 mm	8 weeks (14 days for gait)	Not mentioned	Allogenic	Orthotopic	McClure et al.^32^
Rat: Sprague–Dawley	Gastrocnemius	Complete gastrocnemius muscle	Rat: Sprague–Dawley immunocompetent and RNU immunodeficient	Gastrocnemius	10 × 15 mm	8 weeks (14 days for gait)	immunodeficient verusus immunocompetent rats	Allogenic	Orthotopic	McClure et al.^33^
Rat: Lewis	Gastrocnemius	Complete gastrocnemius muscle	Rat: Lewis	Gastrocnemius	10 × 10 mm	7, 14, 28, and 42 days	Not mentioned	Allogenic	Orthotopic	Merrit et al.^34^
Rat: Sprague–Dawley	Gastrocnemius	Complete gastrocnemius muscle	Rat: RNU (Foxn1RNU)	Gastrocnemius	10 × 15 mm	56 days	immunodeficient	Allogenic	Orthotopic	Olson et al.^35^
Mouse: BALB/c	Tibialis anterior	Complete tibialis anterior muscle	Mouse: BALB/c	Tibialis anterior	Complete muscle	2 and 4 weeks	Cyclosporin A	Allogenic	Orthotopic	Perniconi et al.^36^
Human	Rectus abdominis	15 × 20 mm	Rabbit: New Zealand white	Abdominal muscle: rectus abdominis	15 × 20 mm	3 weeks	Not mentioned	Xenogenic	Orthotopic	Porzionato et al.^37^
Rat: Sprague–Dawley	Tibialis anterior	Not mentioned	Rat: Sprague–Dawley	Tibialis anterior	8 mm diameter biopsy punch, 3 mm deep	3 and 14 days	Not mentioned	Allogenic	Orthotopic	Roberts et al.^38^
Rat: Sprague–Dawley	Intact lower limb	Intact lower limb was decellularized	Mouse: C57BL/6J	Extensor digitorum longus	From distal tendon to half a centimeter from proximal tendon	2 months	Not mentioned	Xenogenic	Heterotopic?	Urciuolo et al.^39^
Rat: Wistar	Rectus abdominis	24 × 8 mm (full thickness)	Rat: Wistar	Abdominal muscle: rectus abdominis	8 × 24 mm (full thickness)	3 weeks	Not mentioned	Allogenic	Orthotopic	Vindigni et al.^40^
Pig: domestic pig	Rectus abdominis	40 × 20 × 5 mm	Rat: Fischer 344	Quadriceps	5 × 10 mm	30 days	Not mentioned	Xenogenic	Heterotopic	Wang et al.^41^
Pig: domestic pig	Triceps surae	50 × 50 × 5 mm	Rat: Sprague Dawley, Rabbit: New Zealand White	Abdominal muscles (full thickness in rabbits and partial thickness in rats)	10 × 15 mm	1, 2, and 4 weeks (rat); 8 weeks (rabbit)	Not mentioned	Xenogenic	Heterotopic	Wang et al.^42^
Dog: Mongrel dog	Whole quadriceps and hamstring	2.25 mm thick sheets	Rat: Sprague–Dawley	Abdominal wall (partial thickness)	10 × 10 mm	14 and 35 days	Not mentioned	Xenogenic	Heterotopic	Wolf et al.^43^
Pig: Yorkshire pigs	Rectus abdominis	Complete distal half below umbilicus	Rat: Sprague–Dawley	Abdominal wall (partial thickness)	15 × 15 mm	2 and 8 weeks	Not mentioned	Xenogenic	Orthotopic	Zhang et al.^44^

Similarly, the implanted DSM grafts were most often derived from rodents: rats in 16 out of 26 studies,^19^, ^20^, ^22^, ^23^, ^25^, ^27^, ^28^, ^30^, ^32^, ^33^, ^34^, ^35^, ^38^, ^39^, ^40^, ^43^ and mice in two studies.^31^, ^36^ However, DSM matrices were also derived from larger donor animals, including rabbit,^24^, ^26^ pig,^41^, ^42^, ^44^ dog^43^ and even human^29^, ^37^ (Figure 3a, Table 2). In 16 out of 26 studies, an allogenic transplantation was performed by implanting a DSM derived from a genetically non‐identical donor of the same species.^19^, ^20^, ^21^, ^22^, ^23^, ^25^, ^27^, ^28^, ^30^, ^32^, ^33^, ^34^, ^35^, ^36^, ^38^, ^40^ In nine out of 26 studies, a xenotransplant was performed where the DSM was derived from a different species.^24^, ^29^, ^31^, ^37^, ^39^, ^41^, ^42^, ^43^, ^44^ One study performed an autogenic transplantation, where the abdominal wall defect was repaired with the decellularized diaphragm of the recipient animal^26^ (Figure 3a, Table 2).

Despite the fact that xenotransplantation and allotransplantation of DSM are the most frequently performed transplantation types, there seems to be no need to apply immunosuppressive therapy upon implantation of the DSM. The majority of the studies (19 out of 26^19^, ^20^, ^21^, ^25^, ^27^, ^28^, ^29^, ^30^, ^31^, ^32^, ^34^, ^37^, ^38^, ^39^, ^40^, ^41^, ^42^, ^43^, ^44^) did not mention the use of any immunosuppressive therapy or immunodeficient animals, and four studies specified that no immunosuppression was used^22^, ^23^, ^24^, ^26^ (Table 2). Importantly, rejection of the DSM was not reported in any of the studies. Only one study reported serious adverse events in which one rat from the implant group died; however, also one rat from the empty defect group died.^35^ Moreover, Zhang et al.^44^ described the presence of seroma in some of the animals at 2 and 8 weeks after the implantation of decellularized rectus abdominis matrices (Table 2). However, seroma formation is a common postoperative complication characterized by the accumulation of serous fluid around the surgical operation site. Provided that it is not infected, seromata are usually harmless and can be treated by placing a drain.

Immunomodulation was applied in two out of 26 studies (Table 2). Perniconi et al.^36^ treated mice with cyclosporine A, which modulates the innate immune system by suppressing T‐cell responses and influencing dendritic cells, macrophages and neutrophils.^52^ Kim et al.^29^ implanted DSM grafts in combination with the administration of nandrolone, an androgenic anabolic steroid that reduces the production of antibodies and induces several inflammatory cytokines.^53^ Results indicated a reduced histological appearance of fibrosis at the repair site in the presence of nandrolone. However, nandrolone did not have a significant benefit on muscle contractility or mass recovery.^29^ Besides the immunomodulatory treatments, immunodeficient RNU (Foxn1^RNU^‐Nude) rats were used by Olson et al.^35^ and McClure et al.^33^ These rats are T‐cell deficient and are often used in transplantation research. Results of McClure et al. suggested improved muscle regeneration when DSM grafts are implanted in immunodeficient rats compared to DSM grafts implanted in their immunocompetent counterparts. Immunohistochemistry demonstrated a decrease in M1 macrophages, no changes in M2 macrophages, an increase in CD4 T‐helper cells, and a reduction in CD8 cytotoxic T‐cells in immunodeficient RNU rats compared to immunocompetent rats. Histological analysis further indicated that the frequency and severity of fibrosis appeared to be greatest in Sprague Dawley rats compared to RNU rats. However, Sprague Dawley rats showed a significantly increased number of regenerated fibers in DSM‐treated sites compared to the empty defect, while this was not the case in RNU rats. Moreover, no differences in tetanic force output were seen between strains, while the twitch force generated by DSM‐treated Sprague Dawley rats was higher than that generated by DSM‐treated RNU rats.^33^

As there are only minimal indications that immunomodulation might be beneficial for skeletal muscle regeneration, this seems favorable for the clinical setting as immunosuppressive treatments hold risks for side effects, including the risk of infections, nephrotoxicity, hypertension, and nervous system disorders.^54^

#### 3.4.2 Implantation of DSM in small‐sized skeletal muscle defects

The skeletal muscle defect was most frequently created in the hindlimb muscles, with eight out of 26 defects in the tibialis anterior muscle,^20^, ^24^, ^27^, ^28^, ^29^, ^30^, ^36^, ^38^ four out of 26 defects in the gastrocnemius muscle,^32^, ^33^, ^34^, ^35^ one defect in the quadriceps muscle,^41^ and one defect in the extensor digitorum longus.^39^ Moreover, nine out of 26 defects were created in the abdominal wall^19^, ^22^, ^23^, ^26^, ^37^, ^40^, ^42^, ^43^, ^44^ and three defects were created in the latissimus dorsi (Figure 3b, Table 2). In 19 out of 26 studies, the skeletal muscle defect was repaired with a DSM derived from the same muscle, that is, orthotopic transplantation,^19^, ^20^, ^21^, ^22^, ^23^, ^24^, ^27^, ^28^, ^29^, ^31^, ^32^, ^33^, ^34^, ^35^, ^36^, ^37^, ^38^, ^40^, ^44^ while the remaining studies performed a heterotopic transplantation where a DSM with a different origin was implanted.^25^, ^26^, ^30^, ^39^, ^41^, ^42^, ^43^ Given the highly organized structure of parallel muscle fibers in skeletal muscle tissue, it is crucial to consider the alignment of the implanted DSM with the muscle fibers of the adjacent recipient muscle. Eight publications explicitly mentioned that the DSM scaffold was implanted in a parallel alignment, while this was not mentioned in the other publications.^20^, ^27^, ^29^, ^32^, ^33^, ^35^, ^36^, ^44^

The size of the skeletal muscle defect in the included studies ranged from a 2‐mm^2^ defect in the abdominal wall^23^ to a complete mouse tibialis anterior muscle^36^ (Table 2). In the majority of the studies, the defect size was around 10 × 10 mm. The size of the created skeletal muscle defect might influence the regeneration efficiency, with smaller defects having a higher degree of spontaneous regeneration. VML exceeding the innate regenerative capacity and resulting in persistent functional impairment is defined as a loss of at least 20% of the muscle mass. Some studies assure the creation of a VML by weighing the excised muscle mass and correlating it to the mean weight of the muscle determined by a previous pilot experiment.^27^, ^28^, ^29^, ^32^ However, from a translational perspective, these defects in small rodent models are still very limited in size (millimeter range). VML injuries in a clinical setting will be of larger dimensions (centimeter range) and might therefore be more challenging to repair with the DSM grafts. Future studies investigating the implantation of DSM matrices in larger defects are crucial to assess the possibility of muscle regeneration, vascularization, and innervation throughout the large‐size defect and assure clinical translatability of this technique.

Another factor influencing the regenerative profile is the follow‐up period. The development of force might be increasing with a longer follow‐up time, while the amount of fibrosis might be adversely correlated with time.^55^ The follow‐up period in rats ranged from 3 days to 3 months, while De Coppi et al.^23^ even analyzed at 9 months after implantation. The follow‐up time in mice was between 12 days and 3 months, and in rabbits, the follow‐up time was between 6 days and 3 months. Different studies showed increased muscle regeneration, including the number of myoblasts and the number of myofibers upon increasing follow‐up time.^22^, ^23^, ^34^ Moreover, vascularization was described to increase over time^22^, ^34^ and Conconi et al.^22^ showed increased functionality over time.

### 3.5 Evaluation of regeneration in DSM upon implantation

No universal scoring system is available to evaluate the regeneration of skeletal muscle defects after implantation of the DSM graft. To provide a comprehensive overview, we divided the analysis of regeneration into four different groups: (i) muscle regeneration, (ii) vascularization of the regenerated muscle, (iii) innervation of the regenerated muscle, and (iv) functional regeneration. In our opinion, these four criteria should be evaluated in future studies to have a complete view of the regenerative potential of DSM grafts.

#### 3.5.1 Assessment of muscle regeneration upon DSM implantation

**FIGURE 4:**
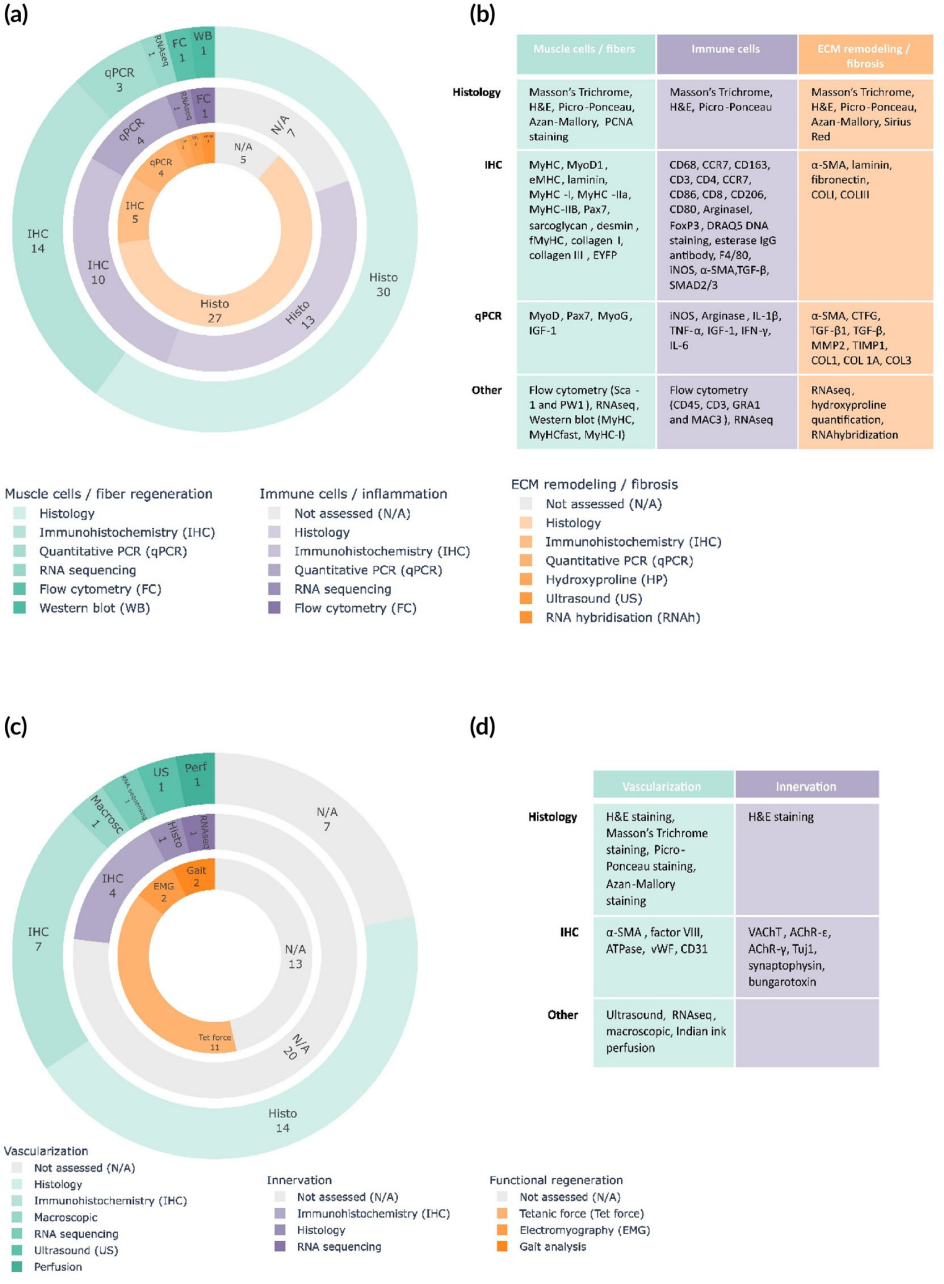
Regeneration of skeletal muscle upon implantation of DSM matrix in skeletal muscle defect. (a) Assessment of muscle regeneration, focusing on muscle cell/fiber generation, immune response, and ECM remodeling. (b) Assessment of vascularization, innervation, and functional regeneration of the regenerated tissue.

All 26 included studies assessed muscle regeneration in the implanted DSM grafts (Figure 4a,b, Table S1). The analysis methods and depth of analysis strongly varied in the different studies. Therefore, we further divided the evaluation of muscle regeneration into three aspects: (i) presence of muscle cells and fibers, (ii) presence of immune cells and inflammation, and (iii) remodeling of the ECM and fibrosis.

All 26 studies evaluated the regeneration of muscle cells and muscle fibers. The most commonly used method for this evaluation is histological staining, including hematoxylin and eosin (H&E), Picro‐Ponceau, Masson's Trichrome, proliferating cell nuclear antigen (PCNA) and Azan‐Mallory staining. Moreover, immunohistochemical stainings for Pax7, desmin, MyoD, fetal MyHC, laminin, embryonic MyHC, MyHC type 1, MyHC type 2A, MyHC type 2B, and sarcoglycan were also performed (Figure 4a,b, Table S1). The presence of muscle cells and fibers at the implantation site is often reported in a descriptive manner, including the morphology, location, orientation, and abundance of cells and fibers in the implant (Table S1). Results of these stainings can also be quantified to obtain the number of cells or fibers and myofiber diameter. Next to the histological evaluation, the presence of key markers for muscle regeneration was also assessed with quantitative PCR to quantify gene expression levels of *MyoD*, *Pax7*, *MyoG*, or *IGF‐1* ^27^, ^28^, ^29^ and with Western blot^35^ to assess protein expression levels of MyHC, MyHCfast, or MyHC‐I in the implanted DSM. Perniconi et al.^36^ showed the presence of muscle stem cells in the graft 2 weeks after implantation using flow cytometry (Sca‐1 and PW1) (Figure 4a,b, Table S1).

Next to the regeneration of muscle cells and myofibers, the infiltration of immune cells and inflammation markers is an important factor in muscle regeneration. Upon muscle injury, an initial inflammatory phase occurs in which M1 macrophages contribute to the phagocytosis of the damaged muscles and the activation of SCs. Later, the macrophages shift to an M2 phenotype, and the regenerative phase is initiated.^3^ Seven out of the 26 included studies did not assess the presence of immune cells or inflammation.^21^, ^23^, ^27^, ^32^, ^34^, ^35^, ^39^ The most frequently used methods are immunohistochemistry (CD68, CD163, CCR7, CD3, CD4, CD80, CD86, ArginaseI, FoxP3, CD206, F4/80, iNOS, CD206, α‐SMA, TGF‐β, and SMAD2/3) and H&E staining. Moreover, quantitative PCR was performed (*iNOS*, *arginase*, *TNF‐α*, *IL‐1β*, *IL‐6*, *IGF‐1*, and *IF‐γ*). Some studies also used Masson's Trichrome and Picro‐Ponceau staining to visualize inflammation and immune cells in the implanted DSM. In addition, Perniconi et al.^36^ assessed the presence of immune cells with flow cytometry (CD45, CD3, GRA1, and MAC3) (Figure 4a,b, Table S1).

Thirdly, remodeling of the implanted ECM and the formation of fibrous tissue were assessed in 21 out of the 26 included publications.^19^, ^20^, ^22^, ^23^, ^25^, ^26^, ^27^, ^28^, ^29^, ^30^, ^31^, ^32^, ^33^, ^35^, ^37^, ^38^, ^40^, ^41^, ^42^, ^43^, ^44^ Histochemical stainings (H&E, Masson's Trichrome, and Ponceau staining) and immunohistochemistry (α‐SMA, laminin, fibronectin, collagen I, and collagen III) followed by a descriptive assessment, were most frequently performed. Moreover, quantitative PCR (*α‐SMA*, *CTGF*, *COL1A*, *MMP2*, *TIMP1*, *COL1*, *COL3*, and *TGFB1*) was performed (Figure 4a,b, Table S1).

In addition to the three above‐mentioned aspects, some studies also reported additional evaluation methods to assess the impact of DSM graft implantation (Table S1). Fifteen studies performed a macroscopic evaluation of the implantation site, describing signs of infections, the size and degree of integration of the DSM, organization of the regenerated muscle, and muscle atrophy. This easy‐to‐perform macroscopic evaluation could serve as a first indication of skeletal muscle regeneration. Interestingly, the muscle mass (normalized to the total body mass and/or the contralateral control) was often reported as a measure for skeletal muscle regeneration. Moreover, ultrasound can be applied to visualize regeneration within the skeletal muscle.^30^, ^35^ Leiva‐cepas et al.^30^ described the angular morphology, injury margins, echogenicity, echotexture, and echostructure of the regenerated muscle, while Olson et al.^35^ described the echogenicity of the formed fibrosis.

#### 3.5.2 Assessment of vascularization of the regenerated tissue

Next to the regeneration of muscle fibers, it is important to assess the degree of vascularization in the regenerated tissue. The presence of a proper vasculature is of great importance in the inflammatory phase of skeletal muscle regeneration to facilitate the recruitment of neutrophils and macrophages and eliminate debris. Moreover, regeneration‐promoting growth factors can be transported to the regenerating tissue.^10^ In addition, the vasculature is crucial to assure the optimal distribution of nutrients and oxygen in the regenerated skeletal muscle tissue.

Vascularization of the regenerated tissue upon implantation of DSM was assessed in 19 out of 26 studies (Figure 4c,d, Table S1).^19^, ^20^, ^21^, ^22^, ^23^, ^24^, ^25^, ^26^, ^30^, ^32^, ^34^, ^37^, ^38^, ^39^, ^40^, ^41^, ^42^, ^43^, ^44^ The most frequently used method is histological staining through H&E, Masson's Trichrome, Picro‐Ponceau, and Azan‐Mallory staining. These general stainings can give a first overview of vascularization within the regenerating tissue. However, they are not specific and might require a trained researcher for proper interpretation. More targeted staining was performed in the included papers with antibodies for α‐SMA, factor VIII, vWF, and CD31. These markers can be used to describe the location, orientation, maturation, and number of blood vessels. In this context, CD31 and vWF are expressed on endothelial cells and thus allow detection of both capillaries and larger blood vessels, while α‐SMA is expressed in the smooth muscle cells present only in larger blood vessels. Of note, none of the included papers used markers for active angiogenesis (e.g., VEGF and CD105). These markers could be an interesting addition in future studies to evaluate the status of the angiogenic process.

Alternatively, vascularization was assessed using ultrasound by Leiva‐cepas et al.^30^ Areas of revascularization were revealed by color Doppler imaging in the tibialis anterior of rats repaired with DSM. The non‐invasive nature of this technique makes it interesting for future studies to evaluate the dynamics of vascularization over time. Lastly, Chen et al.^21^ perfused the regenerated tissue with Indian ink to show a functional anastomosis between the native vasculature of the latissimus dorsi muscle and the vasculature in the implanted DSM.

#### 3.5.3 Assessment of innervation of the regenerated tissue

A third important aspect to assess during the regeneration of skeletal muscle defects is the innervation of the regenerated tissue. Ultimately, innervation is crucial for skeletal muscle contraction and functionality of the regenerated tissue through the release of acetylcholine (ACh) at the neuromuscular junction (NMJ). Moreover, the presence of nerve cells might positively impact the regeneration of the skeletal muscle.^56^

Despite the importance of innervation both for muscle regeneration and functionality, only six out of 26 included studies assessed the innervation of the regenerated tissue (Figure 4c,d, Table S1).^23^, ^32^, ^33^, ^38^, ^39^, ^44^ De Coppi et al.^23^ visualized cholinergic neurons by staining the vesicular acetylcholine transporter (VAChT), a marker for cholinergic neurons in the central and peripheral nervous system.^57^ Results showed VAChT expression on the surface of muscle fibers from 1 month post‐surgery onwards.^23^ Immunofluorescent staining was performed for synaptophysin and bungarotoxin to visualize the presynaptic membrane and the post‐synaptic AChR, respectively. Positive staining for both proteins indicates the presence of a functional NMJ.^35^ To assess the formation of new NMJs and the maturation of the nerves, McClure et al.^32^, ^33^ visualized both embryonic (AChR‐γ) and adult (AChR‐ε) subtypes of the AChR. Results showed that DSM‐repaired skeletal muscle defects stained intensely for embryonic AChR‐γ with little adult AChR‐ε staining.^32^, ^58^ Moreover, class III β‐tubulin (Tuj1) staining was performed to assess the maturation of the nerves.^35^

#### 3.5.4 Assessment of functionality of the regenerated tissue

All above‐mentioned methods assess the regeneration and formation of skeletal muscle tissue on a histological and/or molecular level. However, the ultimate goal of DSM implantation is to regenerate a functional skeletal muscle tissue that allows contraction and voluntary movement for the patient to have fully restored functionality.

Thirteen out of the 26 included studies evaluated functionality upon implantation of DSM in a skeletal muscle defect (Figure 4c,d, Table S1).^21^, ^22^, ^26^, ^27^, ^28^, ^29^, ^32^, ^33^, ^34^, ^35^, ^38^, ^39^, ^42^ The most frequently used technique is to measure the generated tetanic force (11 out of 26 studies^21^, ^27^, ^28^, ^29^, ^32^, ^33^, ^34^, ^35^, ^38^, ^39^, ^42^). For this, the nerve of the anesthetized animal is stimulated ex vivo and the contractility of the muscle is analyzed. While this technique can give valuable information, caution should be taken in the interpretation of the results, in particular for unilateral defects where the non‐operated counterpart serves as control. Due to the unilateral defect, hypertrophy might occur in the contralateral muscle, which can mask improved functionality in the repaired muscle. Therefore, proper normalization of the force measurement to the muscle weight is highly important. In addition, the level of expertise of the operating surgeon could also play a crucial role in the final outcome. Since skeletal muscle tissue is highly aligned, the implantation of DSM into the defect requires meticulous positioning to perfectly fit with the surrounding tissue. Misalignment of the DSM might result in an interruption of the muscle fibers, which negatively influences the force generation. In a study of McClure et al.,^32^ the importance of alignment was demonstrated by repairing a defect in the gastrocnemius of rats with either DSM or a collagen plug. The latter resulted in less muscle regeneration and a lower peak muscle force, which the authors attributed to the lower anisotropy of the collagen plug. Furthermore, the number and thickness of sutures placed in the implanted DSM might also impact the force transmission. Care should be taken to keep these parameters uniform between all operated animals and to restrict the number of sutures to a minimum.

As mentioned above, the most commonly used functional analysis is tetanic force generation and, more specifically, the peak muscle force that can be generated by the implanted DSM. Another interesting approach, however, is to look at muscle fatigue. This approach was proposed by McClure et al.^32^ and enabled the detection of smaller, fatigue‐resistant fibers during submaximal contractions. In this way, more subtle differences between groups could be identified.

When evaluating the repair of muscles of the lower limb, gait analysis can also be performed to gain insight into the locomotion of the animal. McClure et al.^32^, ^33^ analyzed the gait of rats upon DSM implantation in a gastrocnemius defect. The evaluation included external paw rotation, dorsiflexion and plantar flexion angles, hind limb spread, and stance‐to‐swing ratio.^30^, ^31^ This method allows for a more holistic view on muscle regeneration and the interaction with the joints and nervous system, whereas force measurements only give information on the isolated muscle. Moreover, gait analysis is non‐invasive and thus allows for analysis on different timepoints without having to sacrifice the animal.

Lastly, electromyography was performed in two studies to evaluate the electric activity in the regenerated tissue.^22^, ^26^ A thin needle electrode is placed in the center of the implant and a ground electrode is placed in a neutral place (e.g., in the abdomen). Upon stimulation, the muscle will contract and the magnitude of the response can be recorded. In this way, information on the muscle functionality is gained in a minimally invasive manner, which again allows for follow‐up of the same animal over different time points.

### 3.6 Large heterogeneity hampering evaluation of the effectiveness of DSM

Although all 26 included studies assess the implantation of DSM in skeletal muscle defects, it is clear that there is a large variation in the designs of the different studies.

First, no uniform method was applied to create a DSM for implantation. DSM matrices are derived from different muscle origins, and different decellularization and sterilization protocols were applied. Moreover, the implantation experiments largely varied in animal model, transplantation type, and follow‐up time. Furthermore, no power analysis was reported in the design of any of these experiments. Lastly, the assessment of skeletal muscle regeneration upon DSM implantation was not performed in a uniform manner across the different studies. Both the evaluation criteria and the methodology were inconsistent. Only three out of the 26 included studies assessed all four key aspects of regeneration, assessing muscle regeneration, vascularization, innervation, and functional recovery (Table 3). The heterogeneity in the above‐mentioned aspects makes it very challenging to compare different studies regarding the effectiveness of DSM implantation for skeletal muscle regeneration. Therefore, it remains difficult to select the most appropriate DSM grafts that allow successful muscle regeneration.

**TABLE 3:** Overview of analyses performed to assess regeneration and main conclusion of the authors. Presence of assessment of muscle regeneration, vascularization, innervation, or functional recovery upon DSM implantation in the included studies. The main conclusion of the original authors was scored based on a Likert scale (++; +; 0; −; − −) based on the degree to which they agree that DSM matrices form a suitable repair option for skeletal muscle defects.

	Muscle	Vascularization	Innervation	Functional	Likert
Brown et al.^19^	✔	✔			+
Carvalho et al.^20^	✔	✔			+
Chen et al.^21^	✔	✔		✔	++
Conconi et al.^22^	✔	✔		✔	+
De Coppi et al.^23^	✔	✔	✔		++
Fishman et al.^24^	✔	✔			+
Friedrich et al.^25^	✔	✔			+
Gamba et al.^26^	✔	✔		✔	−
Kasukonis et al.^27^	✔			✔	++
Kim et al.^28^	✔			✔	++
Kim et al.^29^	✔			✔	0
Leiva‐Cepas et al.^30^	✔	✔			−
Lin et al.^31^	✔				+
McClure et al.^32^	✔	✔	✔	✔	++
McClure et al.^33^	✔		✔	✔	+
Merrit et al.^34^	✔	✔		✔	+
Olson et al.^35^	✔			✔	+
Perniconi et al.^36^	✔				+
Porzionato et al.^37^	✔	✔			+
Roberts et al.^38^	✔	✔	✔	✔	+
Urciuolo et al.^39^	✔	✔	✔	✔	++
Vindigni et al.^40^	✔	✔			0
Wang et al.^41^	✔	✔			++
Wang et al.^42^	✔	✔		✔	++
Wolf et al.^43^	✔	✔			0
Zhang et al.^44^	✔	✔	✔		++
Total publications	26/26	19/26	6/26	13/26	

*Note:* green means a suitable repair option, red means not suitable.

Since it is very challenging to compare outcomes of the different publications, the authors' conclusions can be used as an indication of the potential of DSM to repair VML. A 5‐step Likert scale was applied to score the degree to which the original authors agree with the statement that DSM forms a suitable repair option for skeletal muscle defects (Table 3). Most authors strongly agree (score: ++) with this statement or agree (score: +) that DSM forms a suitable repair option for skeletal muscle defects, but further research, improvement, or a better study design may improve the outcome. Other authors remain neutral in their opinion due to heterogenic/uncertain results (score: 0).^29^, ^40^, ^43^ Gamba et al.^26^ and Leiva‐cepas et al.^30^ conclude that DSM does not form a suitable repair option for skeletal muscle defects; however, further research, improvement, or a better study design may improve the outcome (score: −). Based on this Likert scale, no author agreed that DSM does not form a suitable repair option for skeletal muscle defects. So, according to the researchers, DSM holds promising potential to repair skeletal muscle defects.

### 3.7 Key points of attention to facilitate clinical translation of DSM

This systematic review highlights a large heterogeneity in ongoing research on the in vivo evaluation of DSM for skeletal muscle repair. To facilitate the translation of DSM to a clinical setting, different points of attention should be considered when performing in vivo studies in the future:

- Optimization of the decellularization methods to increase the size of DSM matrices to obtain grafts of clinically relevant size.
- Implantation of the DSM in skeletal muscle defects in larger animals to evaluate the efficiency of the DSM to regenerate skeletal muscle effects of clinically relevant size.
- Careful consideration of the parallel alignment of the DSM with the adjacent skeletal muscle is necessary to allow optimal regeneration.
- Early consideration of the clinical feasibility of the experimental procedure in patients when designing animal studies (e.g., the use of an autologous cellular component or immunosuppression might not be possible).
- Follow‐up of the regeneration over a longer time to allow adequate assessment of vascularization, innervation, and functionality.
- Thorough, multi‐level investigation of the regeneration by quantitative measures. This evaluation should include:
  - Overall macroscopic (weight, thickness, and color) and microscopic (H&E staining) evaluations of the regenerated tissue.
  - Muscle regeneration is based on the number of myoblasts (desmin staining) and myotubes (MyHC staining), the immune response (CD68, CD3) and the amount of fibrosis (Masson's Trichrome staining).
  - Vascularization of the implanted DSM based on CD31 staining and Indian ink perfusion.
  - Innervation of the DSM implant based on the number of AchR (bungarotoxin staining) and the functionality of the NMJ (synaptophysin staining).
  - Functional evaluation of the regenerated muscle through electromyography or tetanic force measurement.

With this approach, the results of different DSM matrices can be compared, allowing identification of current hurdles and selection of the most optimal DSM matrices for muscle regeneration. Moreover, the upscaling in size to several centimeters will be crucial to assess the feasibility of this technique to regenerate skeletal muscle in defects of clinically relevant size.

### 3.8 Limitations of the study

This review aimed to provide an extensive and complete overview of the state‐of‐the‐art on using DSM for the regeneration of skeletal muscle defects. We carefully considered the search term by including different commonly used terminologies and synonyms to retrieve all relevant literature within our scope.

Cluster analysis of the co‐authors of the included publications revealed 15 different clusters of co‐authors. This indicates that the applied search term was able to retrieve publications from different, unrelated research groups. Nevertheless, the choice of the search term is very delicate, and it might occur that relevant literature is not retrieved because different terminology (e.g., myogenesis instead of regeneration) was used in these publications.

The terms “nerve” and “dermal” were excluded in the search query to limit the number of retrieved papers regarding composite grafts. However, this exclusion could potentially have impacted the number of papers that assess the innervation of DSM upon implantation. Moreover, exclusion of the term “dermal” might have excluded relevant publications comparing DSM with acellular dermal matrices for skeletal muscle regeneration.

## 4 CONCLUSION

DSM holds potential as an implant for the regeneration of skeletal muscle defects by providing the native ECM structure and composition as a blueprint for cell migration and muscle regeneration. In this review, we highlighted the different aspects of study designs and outcomes for all in vivo experiments thus far described. This highlights a large heterogeneity in the ongoing research regarding the creation of DSM matrices, implantation procedures, and the evaluation of the regeneration upon DSM implantation. Moreover, the size of the defects and the implanted DSM matrices remains very limited, urging the need for upscaling in order to progress toward clinically relevant sizes. To accelerate our understanding of the regenerative potential of DSM for skeletal muscle defects, a minimal set of evaluation methods should be implemented, including quantitative evaluation of (i) muscle regeneration, (ii) vascularization of the regenerated muscle, (iii) innervation of the regenerated muscle, and (iv) functional regeneration. Such a structured approach will advance the field as results from several groups worldwide will become more comparable, allowing us to identify current hurdles with DSM implantation and directing further optimization of DSM toward translational applications.

## AUTHOR CONTRIBUTIONS

**Ina Hennion:** Conceptualization; methodology; visualization; writing – original draft; formal analysis; writing – review and editing. **Charlot Philips:** Writing – original draft; methodology; visualization; conceptualization; formal analysis; writing – review and editing. **Chong Jiang:** Conceptualization; methodology; writing – original draft; writing – review and editing. **Nele Van De Winkel:** Writing – original draft; funding acquisition; writing – review and editing. **Laurens J. Ceulemans:** Writing – original draft; funding acquisition; supervision; writing – review and editing. **Lieven Thorrez:** Project administration; writing – original draft; supervision; conceptualization; funding acquisition; writing – review and editing.

## Supporting information


**Table S1:** Methods used to assess skeletal muscle regeneration, vascularization, innervation and functional regeneration.

## Data Availability

The data that support the findings of this study are available from the corresponding author upon reasonable request.
