# Supplementary material for: In vivo evaluation of decellularized skeletal muscle matrices for skeletal muscle repair: A systematic review
Source: Bioeng Transl Med. 2025 Mar 4;10(4):e70009. doi: 10.1002/btm2.70009 (PMC12284429; doi:10.1002/btm2.70009)
Supplement: Supplementary file 1 — Table S1: Methods used to assess skeletal muscle regeneration, vascularization, innervation and functional regeneration. [file BTM2-10-e70009-s001.docx]

**Supplementary table 1: Methods used to assess skeletal muscle regeneration, vascularization, innervation and functional regeneration.**

| Muscle Regeneration | | | | Vascularization | Innervation | Functional regeneration | Reference |
| --- | --- | --- | --- | --- | --- | --- | --- |
| Muscle cells/fibers | **Immune cells/inflammation** | **ECM remodeling/fibrous tissue formation** | **Other** |  |  |  |  |
| Masson's Trichrome staining | Masson's Trichrome staining ; Immunohistochemistry (CD68, CCR7 and CD163) ; qPCR (iNOS and arginase) | Masson's Trichrome staining | N/A | Masson's Trichrome staining | N/A | N/A | Brown et al. (2009) ^19^ |
| H&E, Masson's Trichrome and PCNA staining | H&E and Masson's Trichrome staining; Immunohistochemistry (CD163) | H&E and Masson's Trichrome staining | Macroscopic evaluation (morphology, signs of inflammation, DSM visible, adipose tissue deposition) | H&E staining | N/A | N/A | Carvalho et al. (2021) ^20^ |
| H&E staining; Immunohistochemistry (desmin and MyoD) | N/A | N/A | Macroscopic evaluation (integration, regeneration microvascuature) | Immunohistochemistry (vWF) ; Indian ink perfusion | N/A | Tetanic force measurement | Chen et al. (2013) ^21^ |
| H&E and Picro-Ponceau staining | Picro-Ponceau staining | H&E and Picro-Ponceau staining | N/A | H&E staining ; Picro-Ponceau staining | N/A | Electromyography | Conconi et al. (2004) ^22^ |
| H&E and Picro-Ponceau staining | N/A | H&E and Picro-Ponceau staining | Macroscopic evaluation (dimensions of DSM, retraction) | H&E staining ; Picro-Ponceau staining | Immunohistochemistry (VAChT) | N/A | De Coppi et al. (2006) ^23^ |
| Immunohistochemistry (EYFP) | Immunohistochemistry (CD3, CD4, CCR7, CD163, CD86, Arginase I and FoxP3) | N/A | Macroscopic evaluation (integration) | Immunohistochemistry (α-SMA and factor VIII) | N/A | N/A | Fishman et al. (2013) ^24^ |
| H&E staining | H&E staining ; Immunohistochemistry (CD68, CCR7 and CD206) ; qPCR (IL-1β and TNF‐α) | H&E staining ; Immunohistochemistry (α-SMA) ; qPCR (α‐SMA, CTFG and COL1A) | Macroscopic evaluation (integration, fibrosis formation) | Immunohistochemistry (α-SMA) | N/A | N/A | Friedrich et al. (2018) ^25^ |
| H&E and Picro-Ponceau staining | H&E and Picro-Ponceau staining | H&E and Picro-Ponceau staining | Macroscopic evaluation (DSM size and contraction) | H&E staining ; Picro-Ponceau staining | N/A | Electromyography | Gamba et al. (2002) ^26^ |
| Immunohistochemistry (MHC) ; qPCR (MyoD) | N/A | Masson's Trichrome staining ; Immunohistochemistry (laminin, fibronectin and collagen I and III) ; qPCR (TGF-β1, MMP2, TIMP1, COL1, COL3) | Macroscopic evaluation (infections, gross deformity, muscle size, fibrosis, organisation surface morphology) | N/A | N/A | Tetanic force measurement | Kasukonis et al. (2016) ^27^ |
| Masson's Trichrome staining; Immunohistochemistry (collagen I and III) ; qPCR (MyoD, Pax7, MyoG) | qPCR (TNF-α, IL-1β, IGF-1) | Masson's Trichrome staining ; Immunohistochemistry (collagen I and III) ; qPCR (COL1, COL3, TGFB) | Macroscopic evaluation (muscle atrophy, visibility of the defect) ; muscle mass | N/A | N/A | Tetanic force measurement | Kim et al. (2020) ^28^ |
| Masson's Trichrome staining; Immunohistochemistry (collagen I and III) ; qPCR (MyoD, Pax7, MyoG, IGF-1) | qPCR (TNF-α, IFN-γ, IL-6, IL-1β) | Masson's Trichrome staining; Immunohistochemistry (collagen I and III) ; qPCR (COL1, COL3, TGFB) | Macroscopic evaluation (muscle atrophy), muscle mass | N/A | N/A | Tetanic force measurement | Kim et al. (2022) ^29^ |
| H&E staining | H&E staining | H&E staining | Ultrasound | Immunohistochemistry (ATPase) ; Ultrasound | N/A | N/A | Leiva-cepas et al. (2021) ^30^ |
| H&E staining | H&E staining; Immunohistochemistry (CD68) | H&E staining | N/A | N/A | N/A | N/A | Lin et al. (2014) ^31^ |
| H&E and Masson's Trichrome staining; Immunohistochemistry (Pax7, fMyHC) | N/A | Masson's Trichrome staining | Macroscopic evaluation (muscle crossectional area, gross appearance, adhesions) | H&E and Masson's Trichrome staining | Immunohistochemistry (AChR-ε and AChR-γ) | Tetanic force measurement ; Gait analysis | McClure et al. (2018) ^32^ |
| H&E and Masson's Trichrome staining; Immunohistochemistry (Pax7, fMyHC) | Immunohistochemistry (CD4, CD8, FoxP3, CD68, CD163); DRAQ5 DNA staining | H&E and Masson's Trichrome staining | N/A | N/A | Immunohistochemistry (AChR-ε and AChR-γ) | Tetanic force measurement ; Gait analysis | McClure et al. (2021) ^33^ |
| H&E and Masson's Trichrome staining; Immunohistochemistry (desmin) | N/A | N/A | Muscle mass | Immunohistochemistry (vWF) | N/A | Tetanic force measurement | Merrit et al. (2010) ^34^ |
| Masson's Trichrome staining ; Western blot (MyHC, MyHCfast, MyHC-I) | N/A | Masson's Trichrome staining ; Hydroxyproline quantification ; Ultrasound ; RNA hybridization | RNA hybridization, Western blot and ELISA ( MAP3K, Jun, and cMyc, advanced glycation end-products) | N/A | N/A | Tetanic force measurement | Olson et al. (2022) ^35^ |
| Immunohistochemistry (laminin, MyHC and sarcoglycan) ; Flow cytometry (Sca-1 and PW1) | Immunohistochemistry (esterase and IgG antibody); Flow cytometry (CD45, CD3, GRA1 and MAC3) | N/A | Macroscopic evaluation (size of grafted material) | N/A | N/A | N/A | Perniconi et al. (2011) ^36^ |
| H&E and Azan-Mallory staining | H&E staining | Azan-Mallory staining | Macroscopic evaluation (herniation) | H&E staining ; Azan-Mallory staining | N/A | N/A | Porzionato et al. (2015) ^37^ |
| RNA sequencing | RNA sequencing | RNA sequencing | Macroscopic evaluation (gross appearance, integration) and muscle mass | RNA sequencing | RNA sequencing | Tetanic force measurement | Roberts et al. (2023) ^38^ |
| H&E and Masson's Trichrome staining ; Immunohistochemistry (laminin, MyHC type 1, 2A and 2B) ; Immunohistochemistry (Pax7) | N/A | N/A | Macroscopic appearance (scar tissue, inflammation, tissue regeneration in defect) | Immunohistochemistry (vwf) | Immunohistochemistry (Tuj1, synaptophysin and bungarotoxin) | Tetanic force measurement | Urciuolo et al. (2018) ^39^ |
| Immunohistochemistry (embryonic MyHC) | H&E staining | H&E staining | N/A | Macroscopic evaluation | N/A | N/A | Vindigni et al. (2004) ^40^ |
| Immunohistochemistry (MyoD1) | Immunohistochemistry (CD68, CD80, CD163) | H&E and Masson's Trichrome staining | N/A | Immunohistochemistry (CD31) | N/A | N/A | Wang et al. (2013) ^41^ |
| H&E and Masson's Trichrome staining | Immunohistochemistry (F4/80, iNOS, CD206, α-SMA, TGF-β and SMAD2/3) | H&E and Sirius Red staining; Immunohistochemistry (collagen I and III) | Immunohistochemistry (Myf5 and tenomodulin) | Immunohistochemistry (CD31) | N/A | Tetanic force measurement | Wang et al. (2022) ^42^ |
| Masson's Trichrome staining; Immunohistochemistry (MyHC) | Masson's Trichrome staining | Masson's Trichrome staining | N/A | Masson's Trichrome staining | N/A | N/A | Wolf et al. (2012) ^43^ |
| H&E and Masson's Trichrome staining | H&E staining | H&E staining | Macroscopic evaluation (DSM size) | H&E staining | H&E staining | N/A | Zhang et al. (2016) ^44^ |
